# Agricultural Parameters and Essential Oil Content Composition Prediction of Aniseed, Based on Growing Year, Locality and Fertilization Type—An Artificial Neural Network Approach

**DOI:** 10.3390/life12111722

**Published:** 2022-10-27

**Authors:** Lato Pezo, Biljana Lončar, Olja Šovljanski, Ana Tomić, Vanja Travičić, Milada Pezo, Milica Aćimović

**Affiliations:** 1 Institute of General and Physical Chemistry, University of Belgrade, Studentski trg 12, 11000 Belgrade, Serbia; 2 Faculty of Technology Novi Sad, University of Novi Sad, Bulevar cara Lazara 1, 21000 Novi Sad, Serbia; 3 Department of Thermal Engineering and Energy, “Vinča” Institute of Nuclear Sciences—National Institute of the Republic of Serbia, University of Belgrade, Studentski trg 12, 11000 Belgrade, Serbia; 4 Institute of Field and Vegetable Crops Novi Sad, Maksima Gorkog 30, 21000 Novi Sad, Serbia

**Keywords:** aniseed, essential oil, growing year, locality, fertilization, artificial neural network

## Abstract

**Simple Summary:**

The artificial neural network (ANN) model was developed to predict and optimize the aniseed parameters including: plant height, umbel diameter, number of umbels, number of seeds, 1000-seed weight, yield per plant, plant weight, harvest index, yield per ha, essential oil yield, germination energy, total germination and essential oil content; as well as the content of obtained essential oil, such as: limonene, *cis*-dihydro carvone, methyl chavicol, carvone, *cis*-anethole, *trans*-anethole, β-elemene, α-himachalene, *trans*-β-farnesene, γ-himachalene, *trans*-muurola-4(14),5-diene, α-zingiberene, β-himachalene, β-bisabolene, trans-pseudoisoeugenyl 2-methylbutyrate and epoxy-pseudoisoeugenyl 2-methylbutyrate), according to growing year, locality and fertilization type.

**Abstract:**

Predicting yield is essential for producers, stakeholders and international interchange demand. The majority of the divergence in yield and essential oil content is associated with environmental aspects, including weather conditions, soil variety and cultivation techniques. Therefore, aniseed production was examined in this study. The categorical input variables for artificial neural network modelling were growing year (two successive growing years), growing locality (three different locations in Vojvodina Province, Serbia) and fertilization type (six different treatments). The output variables were morphological and quality parameters, with agricultural importance such as plant height, umbel diameter, number of umbels, number of seeds per umbel, 1000-seed weight, seed yield per plant, plant weight, harvest index, yield per ha, essential oil (EO) yield, germination energy, total germination, EO content, as well as the share of EOs compounds, including limonene, *cis*-dihydro carvone, methyl chavicol, carvone, *cis*-anethole, *trans*-anethole, β-elemene, α-himachalene, *trans*-β-farnesene, γ-himachalene, *trans*-muurola-4(14),5-diene, α-zingiberene, β-himachalene, β-bisabolene, *trans*-pseudoisoeugenyl 2-methylbutyrate and epoxy-pseudoisoeugenyl 2-methylbutyrate. The ANN model predicted agricultural parameters accurately, showing r^2^ values between 0.555 and 0.918, while r^2^ values for the forecasting of essential oil content were between 0.379 and 0.908. According to global sensitivity analysis, the fertilization type was a more influential variable to agricultural parameters, while the location site was more influential to essential oils content.

## 1. Introduction

Anise (*Pimpinella anisum* L.) has been known for centuries as a spice, perfumery and medicinal plant from the *Apiaceae* family [1]. Its fruit is employed in the pharmaceutical industry and daily nutrition due to its vast benefits and ability to mask odors and flavoring [2,3]. The naturally derived compounds are inherently accepted by the human body and are becoming more prevalent as a therapeutic option against various diseases, including viral infections [3]. The characteristic aroma of aniseed arises from the high content of essential oil (3–4%) with *trans*-anethole as the main compound [4]. Aniseeds are frequently applied as an aromatic ingredient in traditional flavored wines [2]. In addition, aniseeds are widely engaged as an aromatic plant to provide flavor to various foods including soups [5], poultry [6], pickles [7], salad [8], drinks [9] and confectionery items, giving them a licorice flavor (chewing gum, jelly beans and candy) [10,11]. It can also be applied as a carminative and sedative agent (due to its antioxidant and antimicrobial properties) [12]. Aniseed can be found in seafood dishes to enhance sweet breath and provide digestive support [12,13]. The essential oils of anise seeds are multicomponent blends of volatile oils, typically terpenes and their derivatives, containing nearly 4% of essential oil (E-Anethole conveys 90% of these essential oils, including stragol, anisaldehyde, γ-himachalene, isoeugenol, anisol, *p*-anisic acid and acetoanisol) [14]. Besides essential oil, aniseeds also contain a significant amount of antioxidants, including phenolic acids and flavonoids [15]. The application of blended fertilizers can significantly improve the biomass and essential oil yield [16]. In addition, the targeted environment and the weather conditions in the growing year can have a substantial impact on essential oil composition [17].

Finding the challenging connection between growing conditions and essential oil composition can be successfully achieved by using mathematical modeling.

The Artificial Neural Network (ANN) was recently recognized as an attractive mathematical method for exploring agricultural production systems [18,19,20]. The ANN model does not require definite model parameters. Nevertheless, it adopts the ability to obtain results from the experimental data and manage the intricate system with nonlinearities and elaborate on the connections between variables [21].

The uses of ANN models cover numerous investigations of agricultural production studies [22]. Lately, the ANN model has been recognized as one of the practical analyses that have been demonstrated to be helpful in drought tolerance indices categorization [23].

The high costs of agricultural production demand to be predicted numerically as much as possible. One of the possible manners of lowering costs is the use of fitting tools that predict agricultural production and variations in kernel properties through breeding. Moreover, the agrotechnology level involved in the cultivation, particularly fertilization with nitrogen, influences seed features that are challenging to prognosticate.

For that reason, the goal of this study was to investigate the probability to accurately predict the plant height, umbel diameter, number of umbels, number of seeds, 1000-seed weight, yield per plant, plant weight, harvest index, yield per ha, EO yield, germination energy, total germination and EO content, as well as the content of obtained EOs, such as limonene, *cis*-dihydro carvone, methyl chavicol, carvone, *cis*-anethole, *trans*-anethole, β-elemene, α-himachalene, *trans*-β-farnesene, γ-himachalene, *trans*-muurola-4(14),5-diene, α-zingiberene, β-himachalene, β-bisabolene, *trans*-pseudoisoeugenyl 2-methylbutyrate and epoxy-pseudoisoeugenyl 2-methylbutyrate throughout agricultural production, as a function of growing year, locality and fertilization type.

In the research by Silitonga et al., [24], the multi-objective optimization (MOO) for adjusting the parameters of agricultural production to maximize the yields was achieved by ANN models associated with ant colony optimization, developed to optimize biodiesel production process parameters.

In accordance with this study, the MOO analysis combined with ANN and genetic algorithm (GA) was implemented in the agricultural process, bearing in mind that there might not be a unique solution due to the contradictory objective functions [18,25,26]. As a part of this study, the solution of the MOO was estimated introducing a Pareto optimal method [18].

## 2. Materials and Methods

### 2.1. Experiment Design

The research was carried out during two successive years at three localities in Vojvodina Province, Serbia (detailed information about locations and soil conditions are given in Table 1). The experiment was carried out in the field under different microclimatic and soil conditions. Field experiments were set up as a randomized block design with four replications. An experimental plot size was 5 m^2^ (consisting of 5 rows, each 3 m long). Sowing was carried out at the optimum time (during April) with a hand seeder. The duration of the vegetation period (in days), as well as climatic conditions, such as growing degree days (GDD), precipitation and insolation, for both investigated years and localities are given in Table 1. Data of meteorological conditions were obtained from the nearest meteorological station for each experimental field (<30 km).

The experiments analyzed the influence of six treatments: control—without fertilizers, Slavol, BactoFil B-10, Royal Ofert, vermicompost and NPK, on different properties of anise. Detailed information about fertilizers is given in Table 2.

The harvest was performed by hand at a full ripening stage. Evaluation of morphological parameters (plant height, umbel diameter, number of umbels, number of seeds, yield per plant, plant weight and harvest index) was performed by sampling 10 randomly selected plants from the central row from each fertilised treatment. Quantity and quality parameters (seed yield per ha, EO yield per ha, EO content, 1000-seed weight, germination energy and total germination) were evaluated by harvesting all plants from three central rows (two outer rows were excluded in order to avoid marginal effect).

### 2.2. ANN Modelling

A multi-layer perceptron model (MLP) with three layers (input, hidden and output) was implemented to construct the ANN model. This format of the ANN model is approved for its high potential to estimate nonlinear functions [27,28,29,30,31].

Prior to the ANN model computation, normalization of input and output data is essential to enhance the outcome of the ANN [32]. Throughout the construction of the ANN model, input data were frequently inserted in the network [31,32,33]. The training process of the network was replicated 100,000 times, testing the various structures of the ANN model, including a diverse number of neurons in the hidden and the output layers (5–20), alternative activation functions (in particular, logarithmic, tangent hyperbolic, logistic or identity), and with random starting values of weight coefficients and biases. The ANN structure optimization was accomplished by achieving the minimal validation error. The BFGS method was implemented to resolve the unconstrained nonlinear optimization problem throughout the ANN construction [34].

The agricultural production database that was employed for the ANN modelling was stochastically segmented into training, cross-validation and testing data (60%, 20% and 20% of experimental data, accordingly). The training data set was applied during the learning cycle of the ANN calculation, the evaluation of the optimal number of neurons in the hidden layer, as well as for the computing of the weight coefficient of individual neurons in the network [35].

The weight coefficients and biases connected to the hidden and output layers of the ANN model are shown in matrices and vectors *W*_1_ and *B*_1_, and *W*_2_ and *B*_2_, individually. The neural network model can be displayed by matrix equation:

(1) $$Y=f_{1}(W_{2}\cdot f_{2}(W_{1}\cdot X+B_{1})+B_{2})$$

where *Y* is the matrix of the outputs, *f*_1_ and *f*_2_ are transfer functions in the hidden and output layers, respectively and *X* is the matrix of inputs [36].

The elements of matrices *W*_1_ and *W*_2_ are computed during the learning cycle, in which the elements are constantly introduced by applying an optimization method to minimize the disagreement between the data and the model [34,37,38]. The BFGS algorithm was implemented to enhance the evaluation and stabilize the solution’s convergence [39]. The coefficients of determination were utilized as parameters to monitor the execution of the achieved ANN model.

The ANN model was created to foresee and optimize the parameters such as plant height, umbel diameter, number of umbels, number of seeds, 1000-seed weight, yield per plant, plant weight, harvest index, yield per ha, EO yield, germination energy, total germination and EO content, as well as the content of obtained EOs, such as limonene, *cis*-dihydro carvone, methyl chavicol, carvone, *cis*-anethole, *trans*-anethole, β-elemene, α-himachalene, *trans*-β-farnesene, γ-himachalene, *trans*-muurola-4(14),5-diene, α-zingiberene, β-himachalene, β-bisabolene, *trans*-pseudoisoeugenyl 2-methylbutyrate and epoxy-pseudoisoeugenyl 2-methylbutyrate, according to growing year, locality and fertilization type.

### 2.3. Global Sensitivity Analysis

Yoon’s global sensitivity formula for the developed ANN model was used to determine the relative influence of the input parameters on output variables, using weight coefficients of the calculated ANN model [40]:

(2) $$RI_{ij}(\%)=\frac{\sum_{k=0}^{n}\left(w_{ik}\cdot w_{kj}\right)}{\sum_{i=0}^{m}\left|\sum_{k=0}^{n}\left(w_{ik}\cdot w_{kj}\right)\right|}\cdot 100\%$$

where *w*—weight coefficient in ANN model, *i*—input variable, *j*—output variable, *k*—hidden neuron, *n*—number of hidden neurons, *m*—number of inputs.

### 2.4. Error Analysis

The numerical confirmation of the developed model was investigated by applying the coefficient of determination (r^2^), reduced chi-square (*χ*^2^), mean bias error (*MBE*), root mean square error (*RMSE*) and mean percentage error (*MPE*). These frequently used parameters can be obtained according to these equations [41]:

(3) $$\chi^{2}=\frac{\sum_{i=1}^{N}{\left(x_{\exp,i}-x_{pre,i}\right)}^{2}}{N-n}$$

(4) $$RMSE={\left[\frac{1}{N}\cdot \sum_{i=1}^{N}{\left(x_{pre,i}-x_{\exp,i}\right)}^{2}\right]}^{1/2}$$

(5) $$MBE=\frac{1}{N}\cdot \sum_{i=1}^{N}\left(x_{pre,i}-x_{\exp,i}\right)$$

(6) $$MPE=\frac{100}{N}\cdot \sum_{i=1}^{N}\left(\frac{\left|x_{pre,i}-x_{\exp,i}\right|}{x_{\exp,i}}\right)$$

where *x_exp_*_,*i*_ marks the experimental values and *x_pre_*_,*i*_ present value computed by the model. *N* and *n* are the number of observations and constants, respectively.

### 2.5. Multi-Objective Optimization

The obtained ANN model was utilized for MOO calculation, with the aim to obtain agricultural production conditions which would reach the maximal values of plant height, umbel diameter, number of umbels, number of seeds, 1000-seeds weight, yield per plant, plant weight, harvest index, yield per ha, EO yield, germination energy, total germination and EO content, as well as the content of obtained EOs, such as limonene, *cis*-dihydro carvone, methyl chavicol, carvone, *cis*-anethole, *trans*-anethole, β-elemene, α-himachalene, *trans*-β-farnesene, γ-himachalene, *trans*-muurola-4(14),5-diene, α-zingiberene, β-himachalene, β-bisabolene, *trans*-pseudoisoeugenyl 2-methylbutyrate and epoxy-pseudoisoeugenyl 2-methylbutyrate. The result of the MOO was extracted using a Pareto front, which appeared in the case of one objective function improvement without deteriorating the others [18]. The genetic algorithm (GA) was used to find the solutions to the MOO problem by a stochastic method inspired by natural evolution in applying the mutation, selection, inheritance and crossover [42,43]. For the MOO computation, Matlab R2018b, software (Gamax Laboratory Solutions Kft., Budapest, Hungary) was used, according to the multi-objective function. The primary population was formed by chance and then introduced to a set of points in the design area. The populations of the next generations were determined using distance measures and a non-dominated ranking of the particular points within the existing generation [18,43,44].

## 3. Results and Discussion

### 3.1. ANN Model

Supplementary Table S1 presents the agricultural parameters of aniseed, based on growing year, locality and fertilization type, while Supplementary Table S2 shows the quantitative profile of *Pimpinella anisum* L. essential oil. The supplementary model was determined by utilizing Equation (1). The attained ANN model showed sufficient generalization ability for experimental data prediction. Based on the ANN model performance, the optimal number of neurons in the hidden layer for obtaining plant height, umbel diameter, number of umbels, number of seeds, 1000-seed weight, yield per plant, plant weight, harvest index, yield per ha, EO yield, germination energy, total germination and EO content, as well as the content of obtained Eos was obtained. The prediction number of neurons in the hidden layer was 10 (network MLP 10-10-30) to attain high values of r^2^ (overall 0.936 for the training period) and as low as a possible sum of squares values (SOS) (Table 3).

**Figure 1:**
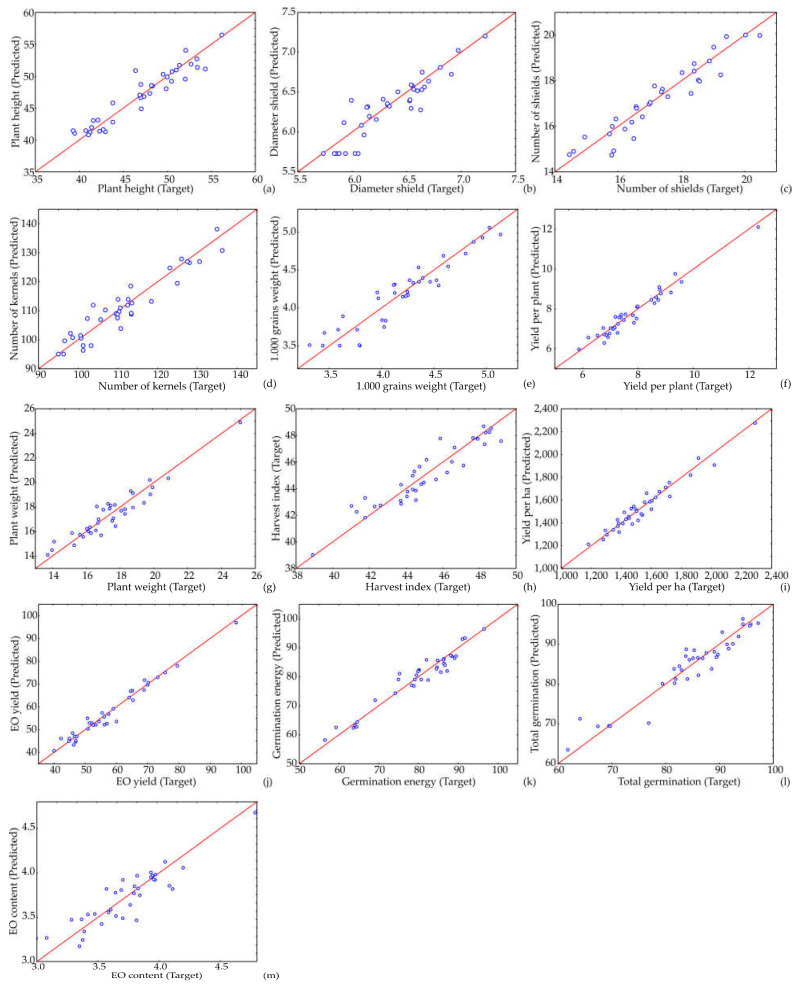
Comparison of experimentally obtained values of (**a**) plant height, (**b**) umbel diameter, (**c**) number of umbels, (**d**) number of seeds, (**e**) 1000-seed weight, (**f**) yield per plant, (**g**) plant weight, (**h**) harvest index, (**i**) yield per ha, (**j**) EO yield, (**k**) germination energy, (**l**) total germination and (**m**) EO content, with ANN predicted values.

**Figure 2:**
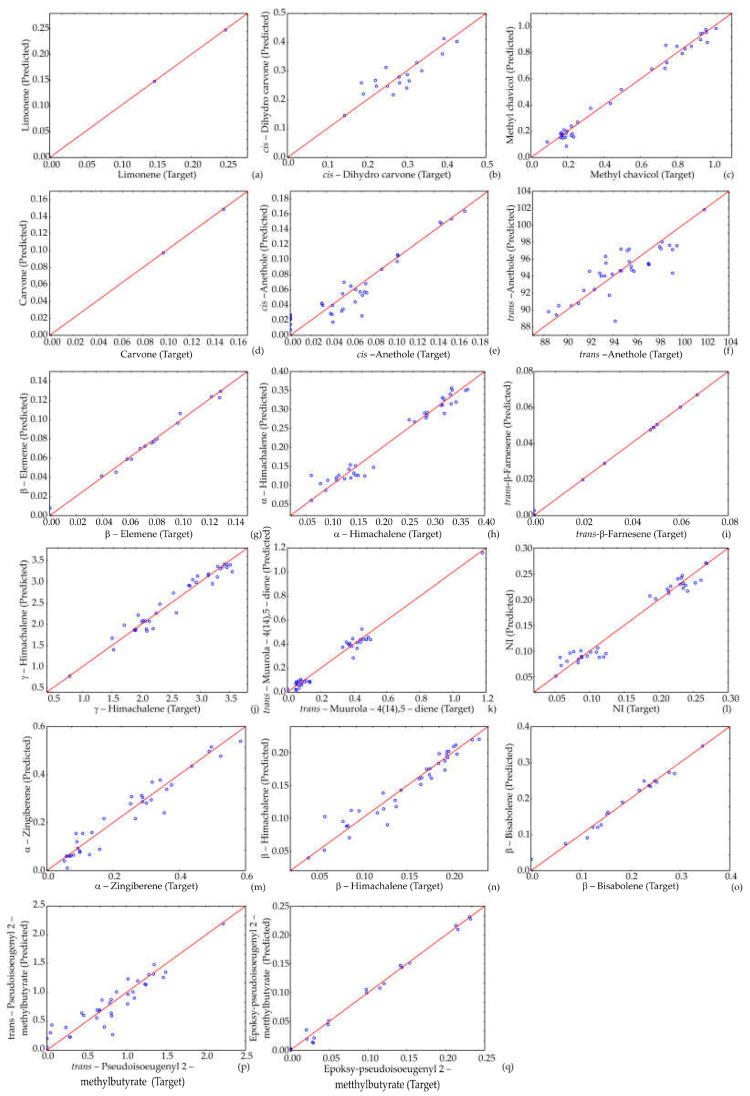
Comparison of experimentally obtained values of (**a**) limonene, (**b**) *cis*-dihydro carvone, (**c**) methyl chavicol, (**d**) carvone, (**e**) *cis*-anethole, (**f**) *trans*-anethole, (**g**) β-elemene, (**h**) α-himachalene, (**i**) *trans*-β-farnesene, (**j**) γ-himachalene, (**k**) *trans*-muurola-4(14),5-diene, (**l**) NI, (**m**) α-zingiberene, (**n**) β-himachalene, (**o**) β-bisabolene, (**p**) *trans*-pseudoisoeugenyl 2-methylbutyrate and (**q**) epoxy-pseudoisoeugenyl 2-methylbutyrate, with ANN predicted values.

Table S3 displays the details of matrix *W*_1_ and vector *B*_1_ (shown in the bias row), and Supplementary Table S4 displays the details of matrix *W*_2_ and vector *B*_2_ (bias) for the hidden layer in the ANN, used for calculation in Equation (1). The ANN model showed an insignificant lack of fit tests, which suggests the model satisfactorily predicted the agricultural parameters and essential oil content composition of aniseed based on growing year, locality and fertilization type. The quality of the model fit was tested, and the residual analysis of the developed model is presented in Table 4 and Table 5. A high r^2^ indicates that the variation was accounted for, and that the data fitted the proposed model satisfactorily [36]. The ANN model was employed to predict experimental variables, quite satisfactorily, for a wide range of the parameters (as observed in Figure 1 and Figure 2, where the experimentally estimated and ANN model predicted values are displayed). 

Most of the time, the predicted values were approaching the desired r^2^ value for the ANN model. Therefore, the SOS achieved by the ANN model is of the same order of magnitude as the experimental errors in Figure 1. Comparison of experimentally obtained values of output variables with ANN predicted values is stated in the articles [34,39,44]. 

The ANN model is challenging (208 weights-biases) due to the high nonlinearity of the studied system [34,45]. The r^2^ values within experimental and ANN model outputs of plant height, umbel diameter, number of umbels, number of seeds, 1000-seed weight, yield per plant, plant weight, harvest index, yield per ha, EO yield, germination energy, total germination and EO content, as well as the content of obtained EOs, such as limonene, *cis*-dihydro carvone, methyl chavicol, carvone, *cis*-anethole, *trans*-anethole, β-elemene, α-himachalene, *trans*-β-farnesene, γ-himachalene, *trans*-muurola-4(14),5-diene, α-zingiberene, β-himachalene, β-bisabolene, *trans*-pseudoisoeugenyl 2-methylbutyrate and epoxy-pseudoisoeugenyl 2-methylbutyrate) were: 0.918; 0.581; 0.893; 0.860; 0.760; 0.828; 0.839; 0.699; 0.826; 0.884; 0.849; 0.861; 0.555; 0.807; 0.908; 0.763; 0.803; 0.578; 0.836; 0.575; 0.893; 0.414; 0.803; 0.665; 0.901; 0.864; 0.631; 0.794; 0.379; and 0.893, respectively, throughout the training period.

The character of the ANN model fit is observed in Table 2 and Table 3, where χ^2^, MBE, RMSE and MPE decrease [41]. The residual analysis of the developed model was additionally conducted. Skewness evaluates the variation of the distribution from normal symmetry. A skewness other than zero indicates the asymmetrical distribution, even though typical distributions are ideally symmetrical. The “peakedness” of distribution is assessed by kurtosis. When the kurtosis is greater than zero, the distribution is flatter or more peaked than predicted; the kurtosis of the normal distribution is zero. A high r^2^ suggests that the variation was evaluated and that the data fit adequately to the suggested model [46,47,48].

The goodness of fit, among experimental computations and model estimated outputs, described as the ANN model performance (sum of r^2^ within measured and calculated parameters), are displayed in Table 3.

### 3.2. Global Sensitivity Analysis—Yoon’s Interpretation Method

**Figure 3:**
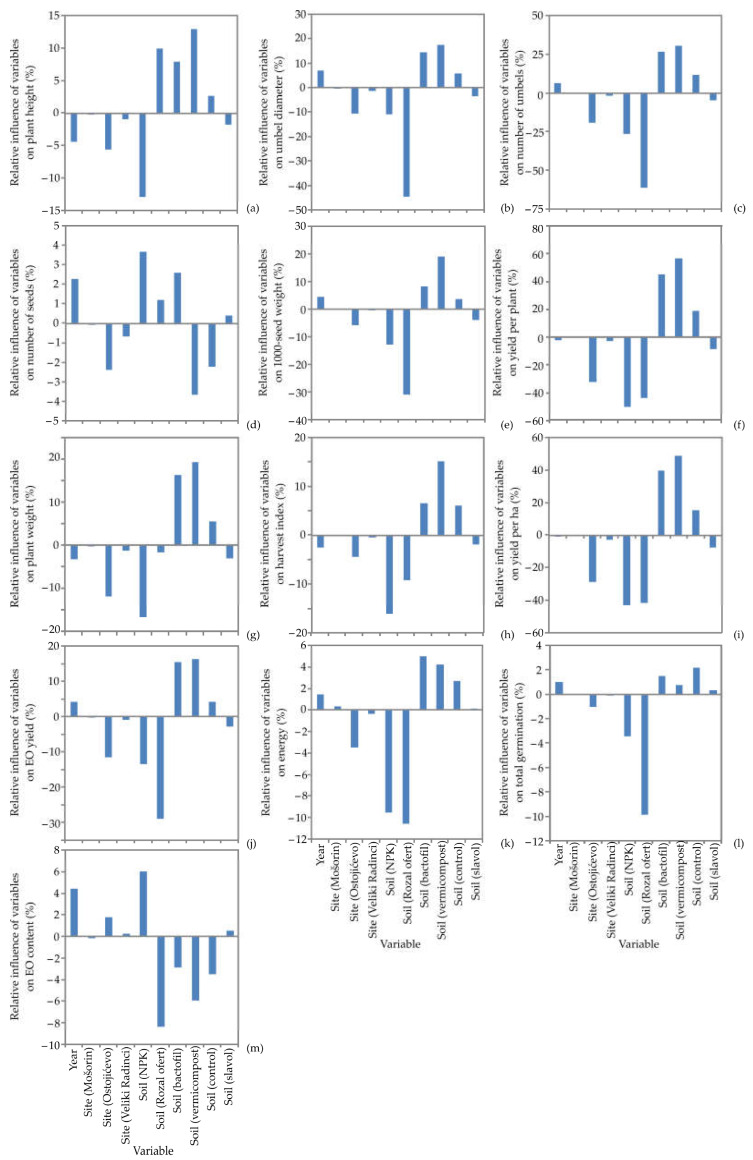
The relative importance of variables on (**a**) plant height, (**b**) umbel diameter, (**c**) number of umbels, (**d**) number of seeds, (**e**) 1000-seed weight, (**f**) yield per plant, (**g**) plant weight, (**h**) harvest index, (**i**) yield per ha, (**j**) EO yield, (**k**) germination energy, (**l**) total germination and (**m**) EO content, determined using Yoon’s interpretation method.

**Figure 4:**
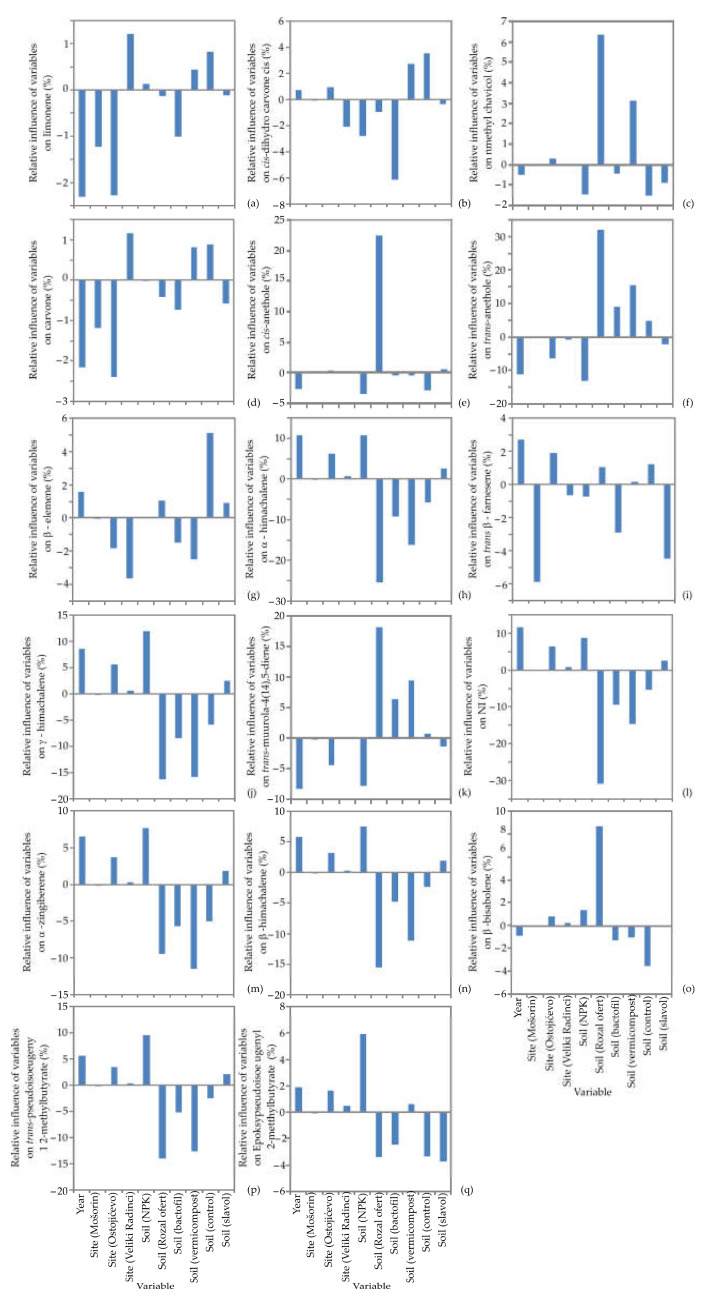
The relative importance of variables on (**a**) limonene, (**b**) *cis*-dihydro carvone, (**c**) methyl chavicol, (**d**) carvone, (**e**) *cis*-anethole, (**f**) *trans*-anethole, (**g**) β-elemene, (**h**) α-himachalene, (**i**) *trans*-β-farnesene, (**j**) γ-himachalene, (**k**) *trans*-muurola-4(14),5-diene, (**l**) NI, m) α-zingiberene, (**n**) β-himachalene, (**o**) β-bisabolene, (**p**) *trans*-pseudoisoeugenyl 2-methylbutyrate and (**q**) epoxy-pseudoisoeugenyl 2-methylbutyrate, determined using Yoon’s interpretation method.

In this segment, the impact of input variables on plant height, umbel diameter, number of umbels, number of seeds, 1000-seed weight, yield per plant, plant weight, harvest index, yield per ha, EO yield, germination energy, total germination and EO content, as well as the content of obtained EOs, such as limonene, *cis*-dihydro carvone, methyl chavicol, carvone, *cis*-anethole, *trans*-anethole, β-elemene, α-himachalene, *trans*-β-farnesene, γ-himachalene, *trans*-muurola-4(14),5-diene, α-zingiberene, β-himachalene, β-bisabolene, *trans*-pseudoisoeugenyl 2-methylbutyrate and epoxy-pseudoisoeugenyl 2-methylbutyrate throughout the agricultural production, Yoon’s interpretation method of a generated ANN model was studied. A detailed illustration of the ANN model results is presented in Figure 3 and Figure 4.

As illustrated in Figure 3, the soil vermicompost and soil BactoFil positively influenced the plant height (relative influences were 12.94% and 7.98%, respectively), umbel diameter (17.69% and 14.35%), number of umbels (26.94% and 25.25%), 1000-seeds weight (19.27% and 8.29%), yield per plant (56.85% and 45.46%), plant weight (19.50% and 16.42%), harvest index (15.30% and 6.59%), yield per ha (48.81% and 39.85%), EO yield (16.36% and 15.62%) and germination energy (5.05% and 4.28%), while the impact of the soil vermicompost on number of seeds (−3.4, %) and EO content (−5.92%) was negative. The most influential parameters negatively affecting 1000-seed weight (−31.18% and −13.93%, respectively), yield per plant (−43.76% and −50.11%), harvest index (−9.16% and −15.95%), yield per ha (−41.44% and −42.86%), germination energy, (−10.62% and −9.59%), EO yield (−29.08% and −13.38%) and total germination (−9.92% and −3.45%) were soil Royal Ofert and soil NPK. Additionally, soil Royal Ofert was the most negatively influential parameter on umbel diameter, number of umbels and EO content, with relative influences of −44.59%, −23.34% and −8.40%, respectively. On the other hand, soil Royal Ofert had a positive influence on the plant height (9.97%). Additionally, soil NPK was the most influential parameter negatively influencing plant height (−12.89%) and plant weight (−16.68%), with quite the opposite trend noticed for number of kernels (3.67%) and EO content (6.05%). According to Figure 3d,f,g,i–k, site Ostojićevo had negative influence on the number of kernels, yield per plant, plant weight, yield per ha, EO yield and germination energy with relative influences of 2.38%, −32.52%, −11.79%, −28.60%, −11.49% and −3.49%, respectively.

It can be determined from Figure 4 that the soil Royal Ofert, soil vermicompost and soil BactoFil were the most negatively influential variables on α-himachalene (relative influences were −16.36%, −15.86% and −8.46%, respectively), NI (−30.87%, −14.64% and −9.44%), α-zingiberene (−9.48%, −11.50% and −5.07%), γ-himachalene (−15.61%, −11.11% and −4.80%), *trans*-pseudoisoeugenyl 2-methylbutyrate (−14.01%, −12.61% and −5.15%), while the impact of the soil Royal Ofert and soil vermicompost on methyl chavicol (6.39% and 3.15%), *trans*-anethole (32.05% and 15.59%) and *trans*-muurola-4(14),5-diene (18.24%, and 9.58%) was positive. Additionally, soil Royal Ofert had a negative influence on β-himachalene (−25.54%) and epoxy-pseudoisoeugenyl 2-methylbutyrate (−3.40%), and a positive influence on *trans*-anethole (22.61%) and β-bisabolene (8.73%. The most influential parameters positively affecting α-himachalene (10.89% and 10.86%, respectively), γ-himachalene (12.05% and 8.63%), β-himachalene (7.53 % and 5.76%), α-zingiberene, (7.72% and 6.47%), NI (8.78% and 11.66%), *trans*-pseudoisoeugenyl 2-methylbutyrate (9.57% and 5.58%) and epoxy-pseudoisoeugenyl 2-methylbutyrate (5.96% and 1.86%), were soil NPK and year, while the opposite trend was noticed for methyl chavicol, carvone, α -anethole and *trans*-muurola-4(14),5-diene. Furthermore, limonene content was negatively influenced by year (−2.32%) and site Ostojićevo (−2.28), and positively influenced by site Veliki Radnici (1.2%) and control soil (0.83%). *Cis*-dihydro carvone content was negatively influenced the most by soil BactoFil (−6.13%) and site Veliki radnici (−2.40%), while control soil showed the opposite influence (3.54%). On the other hand, carvone content was negatively influenced by site Ostojićevo (−2.41%), while positively influenced by site Veliki Radnici (1.16%) and control soil (0.88%). The most influential parameters on β -elemene content were site Veliki radnici (−3.64%) and control soil (5.13%). Finally, *trans*-β farnesene-site content was mostly influenced by site Mošorin (−5.90%), soil Slavol (−4.47%), year (2.73%) and site Ostojićevo (1.91%).

### 3.3. Multi-Objective Optimization of the Outputs of the ANN

One of the main goals of this research was to optimize the developed ANN output variables throughout agricultural production, synchronously employing the ANN model by varying the input variables. These numerical assignments were solved for the ANN model involving the MOO computation in Matlab. The MOO method was set to obtain the most suitable agricultural parameter combinations by maximizing the ANN model’s output variables. Constraints applied to the optimization method were used in the experimental series of parameters. The number of generations achieved was 495 for the ANN model, while the dimension of the population was set to 100 for all input variables. Thus, the number of points on the Pareto front was 232 for the ANN model. The computed maximums of output variables were reached in the first investigated year at Mošorin, without fertilization for harvest index, using Slavol for *cis*-dihydro carvone and *trans*-anethole content; BactoFil for γ-himachalene content; Royal Ofert biohumus for plant height; vermicompost for number of umbels and α-himachalene content; NPK for yield per plant, plant weight, yield per ha, and EO yield, as well as methyl chavicol, *trans*-β-farnesene, α-zingiberene, β-bisabolene and epoxy-pseudoisoeugenyl 2-methylbutyrate content. In the first investigated year at Veliki Radinci, using Royal Ofert biohumus achieved the maximum of output variables for umbel diameter and EO content. During the same year at Ostojićevo, we used fertilizer Slavol for α-himachalene content; BactoFil for 1000-seeds weight, germination energy and total germination; Royal Ofert biohumus for *trans*-β-farnesene content; vermicompost for *cis*-anethole content; and NPK for carvone and limonene content. In the second investigated year, at Veliki Radinci, using NPK, maximums of output variables were reached for the number of seeds. In the same year in Ostojićevo, we used BactoFil for EO compound β-elemene.

The optimal results obtained for plant height, umbel diameter, number of umbels, number of seeds, 1000-seeds weight, yield per plant, plant weight, harvest index, yield per ha, EO yield, germination energy, total germination and EO content, using MO were: 54.389; 6.873; 19.989; 127.251; 5.330; 12.111; 25.683; 50.072; 2281.494; 96.459; 94.752; 96.655; and 4.699, respectively. The optimal content of obtained Eos, such as limonene, *cis*-dihydro carvone, methyl chavicol, carvone, *cis*-anethole, *trans*-anethole, β-elemene, α-himachalene, *trans*-β-farnesene, γ-himachalene, *trans*-muurola-4(14),5-diene, α-zingiberene, β-himachalene, β-bisabolene, *trans*-pseudoisoeugenyl and epoxy-pseudoisoeugenyl 2-methylbutyrate were: 0.242; 0.392; 1.035; 0.154; 0.174; 92.276; 0.126; 0.353; 0.068; 3.590; 1.134; 0.633; 0.240; 0.339; 0.246; and 2.161 respectively.

## 4. Conclusions

According to the presented results, it can be concluded that a developed empirical artificial neural network model could be successfully employed to predict plant height, umbel diameter, number of umbels, number of kernels, 1000-seeds weight, yield per plant, plant weight, harvest index, yield per ha, EO yield, germination energy, total germination and EO content, as well as the content of obtained EOs, such as limonene, *cis*-dihydro carvone, methyl chavicol, carvone, *cis*-anethole, *trans*-anethole, β-elemene, α-himachalene, *trans*-β-farnesene, γ-himachalene, *trans*-muurola-4(14),5-diene, α-zingiberene, β-himachalene, β-bisabolene, *trans*-pseudoisoeugenyl 2-methylbutyrate and epoxy-pseudoisoeugenyl 2-methylbutyrate using year, breeding site and soil type. The artificial neural network analysis delivered a satisfactory fit to observed data and was adequate to predict the output variables successfully, demonstrating a reasonable predictive ability (overall r^2^ for plant height, umbel diameter, number of umbels, number of kernels, 1000-seeds weight, yield per plant, plant weight, harvest index, yield per ha, EO yield, germination energy, total germination and EO content, as well as the content of obtained EOs, such as limonene, *cis*-dihydro carvone, methyl chavicol, carvone, *cis*-anethole, *trans*-anethole, β-elemene, α-himachalene, *trans*-β-farnesene, γ-himachalene, *trans*-muurola-4(14),5-diene, α-zingiberene, β-himachalene, β-bisabolene, *trans*-pseudoisoeugenyl 2-methylbutyrate and epoxy-pseudoisoeugenyl 2-methylbutyrate were 0.918; 0.581; 0.893; 0.860; 0.760; 0.828; 0.839; 0.699; 0.826; 0.884; 0.849; 0.861; 0.555; 0.807; 0.908; 0.763; 0.803; 0.578; 0.836; 0.575; 0.893; 0.414; 0.803; 0.665; 0.901; 0.864; 0.631; 0.794; 0.379; and 0.893, respectively). The developed mathematical model gives satisfactory accuracy for potential practical application in agricultural production.

## Figures and Tables

**Table 1:** Experimental data regarding location.

Location	Ostojićevo	Veliki Radinci	Mošorin
District	Banat	Srem	Bačka
GSM coordinates	45°54′ N, 20°09′ E	45°02′ N, 19°40′ E	45°18′ N, 20°09′ E
Elevation	88 m	111 m	111 m
Soil pH (in KCl)	7.3	7.1	7.3
CaCO_3_ content in the soil (%)	8.8	2.0	8.4
Soil humus (%)	2.2	2.5	2.7
Soil total nitrogen (%)	0.14	0.16	0.18
Soil P_2_O_5_ (mg/100 g)	17.6	22.4	81.6
Soil K_2_O (mg/100 g)	30.3	21.7	71.5
Vegetation period (1st year)	145 days	135 days	133 days
Vegetation period (2nd year)	112 days	118 days	118 days
GDD (1st year)	2752 °C	2413 °C	2350 °C
GDD (2nd year)	2324 °C	2276 °C	2234 °C
Precipitation (1st year)	193 mm	244 mm	191 mm
Precipitation (2nd year)	166 mm	217 mm	183 mm
Insolation (1st year)	1326 h	1041 h	1068 h
Insolation (2nd year)	1115 h	1031 h	1076 h

GDD—growing degree days (measure of heat accumulation during anise vegetation).

**Table 2:** Experimental data regarding treatments.

	Slavol	BactoFil B-10	Royal Ofert	Vermicompost	NPK
Producer	Agrounik, Serbia	BioFil KFT, Hungary	Altamed, Serbia	PG Ivić, Serbia	Elixir Zorka, Serbia
Type	Microbiological		Biohumus		Chemical
Formulation	*Azotobacter chroococcum*, *A. vinelandi*, *Derxia* sp. *Bacillus megaterium*, *B. lichenformis*, *B. subtilis*	*A. vınelandı*, *Azospırıllum brasılense*, *A. lıpoferum*, *B. megaterıum*, *B. suptılıs*, *B. cırkulans*, *B. polymıxa*, *Pseudomonas fluorescens* + natural vitamins and growth stimulator	Made from organic waste from poultry and pig farms inoculated with domestic fly larvae	modified cattle manure with *Lumbricus terrestris*	15:15:15
Application	Watering twice during vegetation	incorporated in the 5 cm layer of soil before the sowing of anise seeds			
Dose	7 L/ha	1.5 L/ha	3 t/ha	5 t/ha	400 kg/ha

**Table 3:** ANN model summary (according to performance and errors) for training, testing and validation cycles.

Net. Name	Performance			Error			Training Algorithm	Error Function	Hidden Activation	Output Activation
	Train	Test	Valid	Train	Test	Valid				
MLP 10-10-30	0.936	0.931	0.925	1141.034	1140.748	1134.330	BFGS 930	SOS	Exp.	Exp.

**Table 4:** The “goodness of fit” tests for the developed ANN model for agricultural data.

	χ^2^	RMSE	MBE	MPE	r^2^	Skew	Kurt	Mean	StDev	Var
Plant height	11.968	1.412	0.090	2.472	0.918	0.645	0.055	0.090	1.429	2.043
Umbel diameter	0.333	0.236	−0.028	3.021	0.581	−0.179	−0.257	−0.028	0.237	0.056
No. of umbels	2.151	0.599	0.046	3.029	0.893	−0.031	−0.883	0.046	0.605	0.367
No. of seeds	89.605	3.864	0.341	2.754	0.860	0.394	−0.164	0.341	3.904	15.241
1.000-seed weight	0.250	0.204	0.027	4.215	0.760	−0.210	−0.636	0.027	0.205	0.042
Yield per plant	1.326	0.470	0.087	4.657	0.828	0.741	0.974	0.087	0.469	0.220
Plant weight	4.466	0.863	0.190	3.883	0.839	0.300	0.038	0.190	0.853	0.728
Harvest index	10.922	1.349	0.044	2.213	0.699	−0.114	0.551	0.044	1.368	1.870
Yield per ha	52,955.718	93.947	14.012	4.709	0.826	0.653	0.841	14.012	94.213	8876.179
EO yield	99.916	4.081	−0.691	6.097	0.884	0.363	0.406	−0.691	4.079	16.638
Germination energy	84.885	3.761	0.443	3.764	0.849	0.456	0.338	0.443	3.788	14.350
Total germination	58.400	3.120	−0.025	2.961	0.861	0.420	0.081	−0.025	3.164	10.011
EO content	0.368	0.247	−0.051	4.744	0.555	−1.543	3.841	−0.051	0.246	0.060

**Table 5:** The “goodness of fit” tests for the developed ANN model for essential oils data.

	χ^2^	RMSE	MBE	MPE	r^2^	Skew	Kurt	Mean	StDev	Var
Limonene	0.004	0.024	−0.011	0.120	0.807	−3.692	16.648	−0.011	0.022	0.000
*cis*-dihydro carvone	0.014	0.048	−0.015	10.138	0.908	0.526	2.739	−0.015	0.046	0.002
Methyl chavicol	0.204	0.184	−0.014	22.759	0.763	−2.592	11.549	−0.014	0.186	0.035
Carvone	0.001	0.015	−0.007	0.240	0.803	−3.878	18.412	−0.007	0.014	0.000
*trans*-anethole	0.006	0.031	0.001	26.468	0.578	−0.930	1.448	0.001	0.031	0.001
*cis*-anethole	3.342	0.746	−0.085	0.624	0.836	0.377	0.286	−0.085	0.752	0.565
β-elemene	0.005	0.029	−0.003	15.886	0.575	0.715	0.989	−0.003	0.029	0.001
α-himachalene	0.007	0.033	−0.004	16.579	0.893	−0.843	1.094	−0.004	0.034	0.001
*trans*-β-farnesene	0.002	0.019	−0.003	16.497	0.414	−1.291	5.865	−0.003	0.019	0.000
γ-himachalene	0.566	0.307	0.007	12.203	0.803	−1.033	1.905	0.007	0.311	0.097
*trans*-muurola-4(14),5-diene	0.151	0.159	−0.023	38.353	0.665	−0.889	3.621	−0.023	0.159	0.025
NI	0.004	0.024	−0.003	15.739	0.901	−0.683	0.538	−0.003	0.025	0.001
α-zingiberene	0.023	0.062	0.006	24.433	0.864	0.362	2.336	0.006	0.063	0.004
β-himachalene	0.006	0.033	0.004	22.162	0.631	0.864	0.829	0.004	0.033	0.001
β-bisabolene	0.018	0.055	−0.010	13.424	0.794	−3.474	15.879	−0.010	0.055	0.003
*trans*-pseudoisoeugenyl 2-methylbutyrate	0.944	0.397	0.020	64.825	0.379	0.954	2.962	0.020	0.402	0.161
Epoxy-pseudoisoeugenyl 2-methylbutyrate	0.004	0.026	−0.006	31.708	0.893	−1.586	4.722	−0.006	0.026	0.001

## Data Availability

Not applicable.

## Supplementary Materials

The supporting information can be downloaded from: https://www.mdpi.com/article/10.3390/life12111722/s1, Table S1: Agricultural parameters of aniseed, based on growing year, locality and fertilization type; Table S2: Quantitative profile of *Pimpinella anisum* L. essential oil (%); Table S3: Details of matrix *W*_1_ and vector *B*_1_; Table S4: Details of matrix *W*_2_ and vector *B*_2_.
