# Supplementary material for: Agricultural Parameters and Essential Oil Content Composition Prediction of Aniseed, Based on Growing Year, Locality and Fertilization Type—An Artificial Neural Network Approach
Source: Life (Basel). 2022 Oct 27;12(11):1722. doi: 10.3390/life12111722 (PMC9694612; doi:10.3390/life12111722)
Supplement: Supplementary file 1 [file life-12-01722-s001.zip › life-1986392-supplementary.pdf]

Table S1. Agricultural parameters of aniseed, based on growing year, locality and fertilization type.

No.	Year	Growing locality	Fertilization type	Plant height (cm)	Umbel diameter (cm)	Number of umbels (№)	Number of seeds (№)	1000-seed weight (g)	Yield per plant (g)	Plant weight (g)	Harvest index	Yield per ha (kg/ha)	Essential oil yield (kg/ha)	Germination energy (%)	Total germination (%)	Essential oil content (%=
1	1st	Mošorin	Control	51.3	5.9	17.0	103.1	4.4	7.5	17.5	48.3	1446.4	56.0	96.4	94.3	4.0
2	1st	Mošorin	Slavol	53.3	6.0	19.2	102.2	4.5	8.5	16.6	49.2	1626.8	65.0	88.3	93.6	4.2
3	1st	Mošorin	Bactofil	52.0	6.6	19.0	100.6	4.3	8.7	17.4	48.6	1692.8	64.5	84.6	84.4	4.0
4	1st	Mošorin	Royal ofert	50.5	6.3	20.5	98.5	4.3	8.8	19.4	48.2	1716.4	69.9	84.8	89.6	3.8
5	1st	Mošorin	Vermicompost	51.0	6.7	20.0	100.6	4.4	8.8	18.8	47.9	1859.2	73.2	86.3	86.9	3.9
6	1st	Mošorin	NPK	56.2	6.2	19.4	127.9	4.5	12.3	25.1	45.1	2286.2	98.3	82.0	91.3	3.9
7	1st	Veliki Radinci	Control	48.3	6.3	15.8	105.5	4.3	6.5	14.1	48.3	1345.9	50.7	78.7	88.5	3.7
8	1st	Veliki Radinci	Slavol	48.0	6.2	15.9	109.5	4.1	7.2	15.6	45.8	1430.5	59.9	78.3	82.5	3.8
9	1st	Veliki Radinci	Bactofil	48.2	6.1	18.5	112.4	4.2	8.6	17.0	47.1	1567.8	70.1	82.4	87.5	4.0
10	1st	Veliki Radinci	Royal ofert	47.0	6.8	18.0	113.3	4.0	8.0	18.1	44.8	1586.2	79.4	74.8	83.8	4.8
11	1st	Veliki Radinci	Vermicompost	50.5	6.9	18.4	113.1	4.0	9.6	19.8	47.6	2015.2	68.8	75.1	85.3	3.6
12	1st	Veliki Radinci	NPK	49.5	6.6	18.9	124.9	4.2	9.3	19.9	47.9	1910.4	75.5	87.1	91.6	4.1
13	1st	Ostojićevo	Control	41.2	6.1	13.7	96.3	4.8	6.8	14.0	44.7	1277.7	46.1	88.1	94.3	3.6
14	1st	Ostojićevo	Slavol	41.0	5.9	13.9	94.9	4.6	6.9	15.3	46.5	1382.6	44.7	86.3	97.1	3.4
15	1st	Ostojićevo	Bactofil	42.1	5.9	16.4	101.2	5.1	7.8	16.6	46.6	1598.4	56.8	91.0	95.8	3.8
16	1st	Ostojićevo	Royal ofert	42.2	5.8	15.7	101.2	5.0	8.0	16.7	48.5	1551.6	58.9	91.6	95.6	3.8
17	1st	Ostojićevo	Vermicompost	41.5	6.1	14.9	98.0	5.0	7.1	16.1	43.7	1510.5	57.4	89.0	92.4	4.0
18	1st	Ostojićevo	NPK	43.8	5.7	16.5	96.5	4.9	7.2	16.7	45.6	1463.0	55.3	89.4	90.5	3.8
19	2nd	Mošorin	Control	50.0	7.2	17.0	110.2	3.8	6.8	16.0	38.9	1375.2	47.2	56.4	61.8	3.6
20	2nd	Mošorin	Slavol	52.1	7.0	18.4	110.5	3.8	7.9	18.3	44.3	1538.7	56.1	64.5	64.0	3.5
21	2nd	Mošorin	Bactofil	52.6	6.5	18.6	109.9	3.4	7.8	17.3	44.0	1497.1	54.4	64.0	76.8	3.4
22	2nd	Mošorin	Royal ofert	53.4	6.3	17.1	113.5	3.6	7.4	18.7	44.5	1418.4	51.9	64.4	69.4	3.7
23	2nd	Mošorin	Vermicompost	54.3	6.1	17.4	112.5	3.8	7.3	17.4	42.3	1499.1	51.3	59.2	67.4	3.3
24	2nd	Mošorin	NPK	46.4	6.5	18.3	118.4	3.3	7.5	18.3	41.0	1531.3	52.4	63.6	69.6	3.8
25	2nd	Veliki Radinci	Control	49.8	5.9	16.2	123.0	3.5	6.2	16.9	41.8	1303.3	45.9	69.0	84.0	3.8
26	2nd	Veliki Radinci	Slavol	47.3	6.6	16.8	130.5	3.6	7.3	17.6	41.3	1408.0	50.5	74.2	86.0	3.7

27	2nd	Veliki Radinci	Bactofil	47.0	6.6	16.6	126.0	3.6	7.4	17.7	43.7	1481.4	53.4	84.5	89.0	3.6
28	2nd	Veliki Radinci	Royal ofert	47.0	6.7	16.6	127.4	4.0	8.7	18.6	44.5	1602.9	65.2	80.1	83.6	4.1
29	2nd	Veliki Radinci	Vermicompost	43.8	6.5	17.6	136.2	4.0	8.8	19.7	41.7	1651.6	64.0	85.5	89.4	3.6
30	2nd	Veliki Radinci	NPK	46.8	6.3	17.4	134.9	4.0	9.2	20.9	42.6	1721.6	68.7	79.1	85.1	3.9
31	2nd	Ostojićevo	Control	39.4	6.1	13.7	110.6	4.6	5.9	13.7	44.0	1177.7	39.9	80.0	81.5	3.3
32	2nd	Ostojićevo	Slavol	41.4	6.4	13.9	106.8	4.2	6.8	15.8	44.4	1292.6	44.8	86.2	86.0	3.5
33	2nd	Ostojićevo	Bactofil	40.7	6.5	14.5	113.3	4.4	7.0	15.2	46.2	1380.0	46.9	86.6	82.9	3.4
34	2nd	Ostojićevo	Royal ofert	42.9	6.6	15.8	109.8	4.5	7.3	16.3	44.9	1470.9	46.9	79.4	81.6	3.0
35	2nd	Ostojićevo	Vermicompost	39.3	6.6	14.6	109.9	4.1	6.7	16.2	44.3	1448.7	42.2	79.9	81.9	3.1
36	2nd	Ostojićevo	NPK	42.7	6.0	15.9	103.7	4.1	7.0	17.7	43.7	1374.3	46.6	80.7	79.4	3.4

Table S2. Quantitative profile of *Pimpinella anisum* L. essential oil (%).

No	Limonene	*cis*-dihydro carvone	Methyl chavicol	Carvone	*cis*-anethole	*trans*-anethole	β-elemene	α-himachalene	*trans*-β-farnesene	γ-himachalene	*trans*-muurola -4(14).5-diene	NI	α-zingiberene	β-himachalene	β-bisabolene	*trans*-pseudoisoeugenyl 2-methylbutyrate	*epoksy*-pseudoisoeugenyl 2-methylbutyrate
1	0.0	0.0	0.7	0.0	0.0	94.2	0.0	0.3	0.0	2.9	0.3	0.2	0.3	0.2	0.2	1.5	0.2
2	0.0	0.0	1.0	0.0	0.1	88.4	0.0	0.3	0.0	3.5	0.4	0.2	0.4	0.2	0.3	1.0	0.1
3	0.0	0.0	1.0	0.0	0.0	93.7	0.0	0.3	0.0	3.3	0.4	0.2	0.5	0.2	0.2	1.2	0.1
4	0.0	0.0	0.9	0.0	0.0	91.0	0.0	0.3	0.1	3.4	0.4	0.2	0.5	0.2	0.3	1.3	0.2
5	0.0	0.0	0.7	0.0	0.1	89.2	0.0	0.4	0.0	3.5	0.4	0.3	0.5	0.2	0.3	1.1	0.2
6	0.0	0.0	1.0	0.0	0.1	89.1	0.0	0.3	0.1	3.4	0.4	0.2	0.6	0.2	0.3	1.3	0.2
7	0.0	0.0	0.9	0.0	0.1	91.4	0.0	0.3	0.0	2.8	0.4	0.2	0.3	0.2	0.1	0.8	0.0
8	0.0	0.0	0.9	0.0	0.1	97.0	0.0	0.3	0.0	3.2	0.5	0.2	0.3	0.2	0.2	0.8	0.0
9	0.0	0.0	0.8	0.0	0.1	93.3	0.0	0.3	0.0	2.9	0.4	0.2	0.3	0.2	0.1	0.7	0.0
10	0.0	0.0	1.0	0.0	0.1	97.0	0.0	0.3	0.1	3.1	0.5	0.2	0.4	0.2	0.2	1.0	0.1
11	0.0	0.0	0.8	0.0	0.1	97.1	0.0	0.3	0.0	2.9	0.4	0.2	0.3	0.2	0.1	0.6	0.0
12	0.0	0.0	0.8	0.0	0.1	92.9	0.0	0.3	0.0	2.8	0.4	0.2	0.3	0.2	0.1	0.4	0.0
13	0.0	0.0	0.3	0.0	0.1	90.3	0.0	0.3	0.0	3.3	0.5	0.3	0.4	0.2	0.1	0.9	0.1
14	0.0	0.0	0.4	0.0	0.1	94.5	0.0	0.4	0.0	3.5	0.4	0.3	0.3	0.2	0.2	1.1	0.1
15	0.0	0.0	0.5	0.0	0.0	95.5	0.0	0.3	0.0	3.3	0.4	0.2	0.2	0.2	0.0	0.7	0.0
16	0.1	0.0	0.7	0.1	0.2	94.7	0.0	0.3	0.0	2.5	1.2	0.2	0.4	0.1	0.2	0.7	0.0
17	0.0	0.0	0.9	0.0	0.2	93.2	0.0	0.3	0.0	3.1	0.4	0.2	0.3	0.2	0.2	0.7	0.1
18	0.2	0.0	0.7	0.1	0.1	92.3	0.0	0.3	0.0	3.4	0.5	0.3	0.3	0.2	0.2	1.0	0.2
19	0.0	0.1	0.2	0.0	0.0	101.9	0.0	0.1	0.0	0.8	0.0	0.1	0.0	0.0	0.0	0.0	0.0
20	0.0	0.4	0.2	0.0	0.0	95.4	0.0	0.1	0.0	2.1	0.1	0.1	0.1	0.1	0.0	0.8	0.0
21	0.0	0.2	0.2	0.0	0.0	95.2	0.1	0.1	0.0	1.9	0.1	0.1	0.1	0.2	0.0	0.5	0.0
22	0.0	0.3	0.2	0.0	0.1	95.4	0.1	0.1	0.0	1.7	0.1	0.1	0.1	0.1	0.0	0.0	0.0
23	0.0	0.3	0.2	0.0	0.0	99.5	0.1	0.2	0.0	2.1	0.1	0.1	0.1	0.1	0.0	0.3	0.0
24	0.0	0.2	0.2	0.0	0.0	98.9	0.1	0.1	0.0	2.2	0.1	0.1	0.1	0.1	0.0	0.3	0.0
25	0.0	0.2	0.2	0.0	0.0	95.6	0.1	0.1	0.0	2.1	0.1	0.1	0.1	0.1	0.0	0.8	0.0

26	0.0	0.3	0.2	0.0	0.1	95.8	0.1	0.1	0.0	1.9	0.1	0.1	0.1	0.1	0.0	0.1	0.0
27	0.0	0.2	0.2	0.0	0.0	91.9	0.0	0.1	0.0	2.0	0.0	0.1	0.1	0.1	0.0	1.2	0.0
28	0.0	0.3	0.2	0.0	0.1	92.8	0.1	0.1	0.0	2.0	0.1	0.1	0.1	0.1	0.0	0.8	0.0
29	0.0	0.3	0.2	0.0	0.1	99.1	0.0	0.1	0.0	1.9	0.1	0.1	0.1	0.1	0.0	0.6	0.0
30	0.0	0.3	0.2	0.0	0.1	93.9	0.1	0.2	0.0	2.1	0.0	0.1	0.1	0.1	0.0	1.1	0.0
31	0.0	0.4	0.2	0.0	0.0	98.2	0.1	0.1	0.0	2.3	0.1	0.1	0.2	0.2	0.0	2.2	0.0
32	0.0	0.4	0.3	0.0	0.1	93.3	0.1	0.2	0.0	2.6	0.1	0.1	0.3	0.2	0.0	1.3	0.0
33	0.0	0.3	0.2	0.0	0.0	99.1	0.1	0.1	0.0	2.2	0.1	0.1	0.1	0.1	0.0	1.5	0.0
34	0.0	0.3	0.2	0.0	0.0	98.0	0.0	0.1	0.0	1.5	0.1	0.1	0.1	0.1	0.0	0.0	0.0
35	0.0	0.2	0.2	0.0	0.0	98.1	0.1	0.1	0.0	1.9	0.1	0.1	0.1	0.1	0.0	0.2	0.0
36	0.0	0.3	0.1	0.0	0.0	94.6	0.0	0.1	0.0	1.5	0.1	0.1	0.1	0.1	0.0	0.0	0.0

Table S3. Details of matrix W_1 and vector B_1.

	1	2	3	4	5	6	7	8	9	10
Year	0.979	0.946	-1.522	-0.214	3.365	0.880	-2.116	5.128	0.999	5.250
Location(MOŠORIN)	0.956	-3.762	1.878	-4.751	1.318	0.178	1.749	2.617	-4.022	2.739
Location(OSTOJIĆEVO)	2.029	1.475	0.614	4.165	0.220	1.560	0.704	1.353	3.147	1.230
Location(VELIKI RADINCI)	-1.921	1.912	0.079	1.222	-2.375	-1.309	-0.197	-6.902	0.800	-6.052
Fertilization type (NPK)	0.013	0.722	0.208	-0.039	-0.199	-0.101	0.193	-0.597	-0.182	-0.438
Fertilization type (Royal ofert)	0.043	-1.282	0.313	-0.012	-0.222	-0.072	0.291	-0.580	-0.157	-0.402
Fertilization type (bactofil)	0.131	0.859	0.411	0.062	-0.136	-0.003	0.389	-0.490	-0.082	-0.308
Fertilization type (vermicompost)	0.045	0.958	0.293	-0.024	-0.234	-0.076	0.272	-0.594	-0.171	-0.420
Fertilization type (kontrola)	0.478	-1.201	0.728	0.402	0.183	0.348	0.709	-0.211	0.259	-0.092
Fertilization type (slavol)	0.291	-0.438	0.536	0.223	-0.130	0.163	0.515	-0.481	0.078	-0.342
Bias	1.059	-0.274	2.520	0.591	-0.797	0.382	2.355	-2.802	-0.105	-2.102

Table S4. Details of matrix W_2 and vector B_2.

	1	2	3	4	5	6	7	8	9	10	Bias
Plant height	-0.228	-0.011	-0.294	-0.047	-0.680	0.526	0.421	0.682	0.141	-0.090	0.151
Umbel diameter	0.280	-0.013	-0.427	-0.048	-0.428	-1.771	0.570	0.703	0.233	-0.144	1.619
No. of umbels	0.144	0.000	-0.432	-0.038	-0.603	-1.394	0.602	0.689	0.269	-0.102	0.772
No. of seeds	0.161	-0.003	-0.166	-0.046	0.255	0.085	0.181	-0.253	-0.153	0.028	0.863
1.000-seeds weight	0.204	0.002	-0.260	-0.007	-0.571	-1.377	0.366	0.851	0.159	-0.178	-0.104
Yield per plant	-0.083	0.007	-1.149	-0.106	-1.771	-1.546	1.607	2.009	0.664	-0.298	1.851
Plant weight	-0.330	0.005	-1.195	-0.121	-1.691	-0.158	1.665	1.977	0.559	-0.298	1.754
Harvest index	-0.258	0.005	-0.451	-0.037	-1.658	-0.952	0.685	1.591	0.643	-0.197	-0.286
Yield per ha	-0.025	0.007	-1.083	-0.100	-1.623	-1.569	1.509	1.848	0.595	-0.277	1.825
EO yield	0.218	-0.004	-0.595	-0.050	-0.693	-1.506	0.809	0.847	0.214	-0.143	1.541
Germination energy	0.041	0.009	-0.100	-0.009	-0.273	-0.303	0.144	0.122	0.077	0.004	-0.405
Total germination	0.074	0.003	-0.074	-0.005	-0.252	-0.724	0.113	0.058	0.160	0.025	-0.294
EO content	0.408	-0.013	0.162	0.022	0.552	-0.768	-0.264	-0.541	-0.320	0.051	0.246
Limonene	-8.940	-4.731	-8.802	4.694	0.517	-0.503	-3.926	1.700	3.219	-0.458	2.877
cis-dihydro carvone	0.422	-0.009	0.546	-1.210	-1.589	-0.528	-3.526	1.565	2.036	-0.196	0.363
Methyl chavicol	-0.368	-0.011	0.185	0.011	-0.989	4.303	-0.296	2.123	-1.036	-0.614	0.357
Carvone	-9.267	-5.094	-10.306	4.963	-0.015	-1.752	-3.143	3.510	3.760	-2.445	3.835
trans-anethole	-2.506	-0.036	0.304	-0.178	-3.217	20.799	-0.560	-0.467	-2.747	0.540	-0.072
cis-anethole	-0.812	0.009	-0.462	-0.056	-0.950	2.307	0.661	1.122	0.337	-0.155	-1.251
β-elemene	3.619	-0.062	-4.147	-8.333	0.142	2.363	-3.387	-5.682	11.743	2.022	0.780
α-himachalene	1.518	-0.010	0.874	0.098	1.523	-3.572	-1.294	-2.269	-0.812	0.377	0.201
trans-β-farnesene	10.054	-21.725	7.034	-2.418	-2.713	3.868	-10.734	0.672	4.560	-16.468	26.778
γ-himachalene	0.902	-0.007	0.595	0.063	1.260	-1.711	-0.885	-1.659	-0.615	0.258	-0.043
trans-Muurola-4(14).5-diene	-2.503	-0.048	-1.332	0.034	-2.358	5.589	1.981	2.934	0.226	-0.400	-0.671
NI	1.046	-0.015	0.576	0.071	0.788	-2.770	-0.847	-1.313	-0.485	0.225	0.106
α-zingiberene	1.302	-0.030	0.750	0.067	1.553	-1.908	-1.142	-2.316	-1.002	0.382	0.419
β-himachalene	1.388	-0.026	0.783	0.084	1.815	-3.763	-1.157	-2.679	-0.547	0.484	0.064
β-bisabolene	-1.039	-0.055	1.018	0.358	1.691	10.507	-1.528	-1.239	-4.182	-0.028	0.032

trans-pseudoisoeugenyl 2-methylbutyrate	3.612	-0.002	2.255	0.234	6.196	-9.065	-3.332	-8.163	-1.661	1.391	-0.983
Epoksy-pseudoisoeugenyl 2-metthylbutyrate	4.098	-0.119	3.636	1.027	13.025	-7.437	-5.346	1.316	-7.370	-8.247	1.234
